# Polyamine-Promoted Growth of One-Dimensional Nanostructure-Based Silica and Its Feature in Catalyst Design

**DOI:** 10.3390/ma5101787

**Published:** 2012-10-01

**Authors:** Xin-Ling Liu, Pei-Xin Zhu, Yan-Feng Gao, Ren-Hua Jin

**Affiliations:** 1 Shanghai Institute of Ceramics, Chinese Academy of Sciences, 1295 Dingxi Road, Shanghai 200050, China; E-Mails: lxl081115@gmail.com (X.-L.L.); gaosic@gmail.com (Y.-F.G.); 2 Synthetic Chemistry Lab., Kawamura Institute of Chemical Research, 631 Sakado, Sakura 285-0078, Japan; E-Mail: zhu@kicr.or.jp; 3 Department of Material and Life Chemistry, Faculty of Engineering, Kanagawa University and JST-CREST, 3-2-7 Rokkakubashi, Yokohama 221-8686, Japan

**Keywords:** biomimetic silicification, polyethyleneimine, crystalline aggregates, nanostructured silica, silica nanotube, silica nanowire

## Abstract

Crystalline linear polyethyleneimine (LPEI) is a fascinating polymer that can be used as a catalyst, template and scaffold in order to direct the formation of silica with controllable compositions and spatial structures under mild conditions. Considering the crystallization and assembly of LPEI is temperature-dependent, we adopted different accelerated cooling processes of a hot aqueous solution of LPEI in order to modulate the LPEI crystalline aggregates. We then used them in the hydrolytic condensation of alkoxysilane. A series of silica with nanofibrils, nanotubes and nanowire-based structures were achieved simply by the LPEI aggregates which were pre-formed in defined cooling processes. These specific one-dimensional nanoscale structures assembled into microscale fibers-, sheet- and platelet-like coalescences. Furthermore, the deposition kinetics was also researched by the combination of other characterizations (e.g., pH measurement, ^29^Si MAS NMR). As a preliminary application, the hybrids of LPEI@SiO_2_ were used not only as an agent for reducing PtCl_4_^2−^ into Pt but also as host for loading Pt nanoparticles. The Pt-loaded silica showed good catalytic properties in the reduction of Rhodamine B by dimethylaminoborane (DMAB).

## 1. Introduction

Tailoring materials on controlled architectures and compositions is of lasting importance for materials scientists since the properties and performance of materials are strongly dependent upon their structured compositions. One route to approach a tailor-made goal is so-called biomimicry: learning from nature to design and control chemical reactions and domain growths. In nature, silica in diatoms and sponges possess fascinating and diverse patterns on many length scales, which inspires scientists to develop the so-called bioinspired/biomimetic synthetic strategies [1,2,3,4,5].

Accumulated knowledge on biosilicification has revealed that a family of long-chain polyamines in organisms plays several key roles in the fabrication of silica: (i) Long-chain polyamines self-assemble into defined structures to act as templates and scaffolds for directing the spatial structure of silica; (ii) the specific functional groups contained in the long-chain polyamines can promote the hydrolysis and condensation of silica source and the subsequent aggregation of silica species. Taking these considerations into biomimetic synthesis, two facets are worthy for probing: One is to design and synthesize long-chain polyamines analogue polymers and the other is to manipulate their assembly manners in various ways. A lot of work in this respect has been reported in the past ten years [6,7,8,9,10,11,12,13,14]. As a unique biomimetic approach, we have established a linear polyethyleneimine (LPEI)-based methodology to construct nanostructured silica and titania under mild conditions. Due to its crystalline nature of LPEI, 1D structure-based silica materials could be easily obtained in a two-step process. Namely, the crystalline LPEI aggregates were firstly obtained by naturally cooling hot aqueous solutions of LPEI to room temperature, and silica deposited on the preorganized LPEI aggregates after the addition of a silica source. In our method, developing LPEI crystalline assemblies with spatial characteristics is a key step because both the temporal issue of the sol-gel reaction and the spatial issue of the product morphologies are controlled by the LPEI assemblies (templates). Therefore, tailoring the architecture of silica with special structures needs to modulate the LPEI crystalline aggregates that control the silica formation temporally and spatially. We have found many simple factors such as solvents, pH, concentrations and additives to modulate LPEI aggregates from aqueous solution during the natural cooling process [15,16,17,18,19,20,21,22,23,24]. In spite of the differences on the parameters based in these modulations, a common point was shared. That is, LPEI crystallized and grew maturely into fibrous bundles via which were lack in 1D-structure with well-controlled length and width in the nanoscale.

To overcome some shortcomings of our previous methods and to achieve the diverse technique of sophisticated silica processing seen in biological systems, we further attempted to develop controllable and scalable approaches for modulating 1D structure-based LPEI aggregates and their directing silica formation. Since the crystallization of LPEI is temperatures-dependent, it was expected that nucleation and growth of crystalline LPEI aggregates should be controlled by the temperature change in the cooling process. To confirm this idea, several accelerated cooling processes different from the previous natural cooling was chosen and conducted by the direct addition of given amounts of the crushed ice to hot aqueous solution of LPEI. Indeed, LPEI@SiO_2_ with tunable sizes and morphologies (*i.e.*, fibrils, tubes, wires) in the nano-dimensions could be easily scalable (gram-scale) under different cooling processes, and the morphologies in the micro-scale were correspondingly changed. Furthermore, the modulation of the amount of LPEI and silica source was employed to not only control the compositions, porosity, and surface areas but also to understand some details of the biomimetic silicification. Additionally, a preliminary feature of the silica in catalysis was also explored by loading Pt nanoparticles on LPEI@SiO_2_.

## 2. Results and Discussion

To achieve an accelerated cooling process, crushed ice was employed to control the temperature change. As shown in Table 1, four typical accelerated cooling processes named with Process-I, -II, -III and -IV were introduced by the addition of 4, 10, 25 and 50 g of ice, respectively. After addition of crushed ice to the hot LPEI solution, the temperature of the mixture decreased quickly in a short time. For Process-I, the temperature decreased rapidly from 85 °C to about 39 °C in 1 min and then slowly decreased to room temperature; for Process-II, it reached the lowest temperature of about 12 °C in 1 min; for Process -III and -IV, it decreased the lowest temperature of 1 °C in 1 min.

**Table 1:** Temperature changes after addition of crushed ice.

	Process-I	Process-II	Process-III	Process-IV
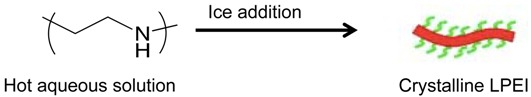				
linear polyethyleneimine (LPEI) wt % (Amount)	2, 5, 10 (10 g)	2, 5, 10 (10 g)	2, 5, 10 (10 g)	2 (10 g)
Amount of ice added (g)	4	10	25	50
The lowest temperature after addition of ice	39 °C	12 °C	1 °C	1 °C

**Figure 1:**
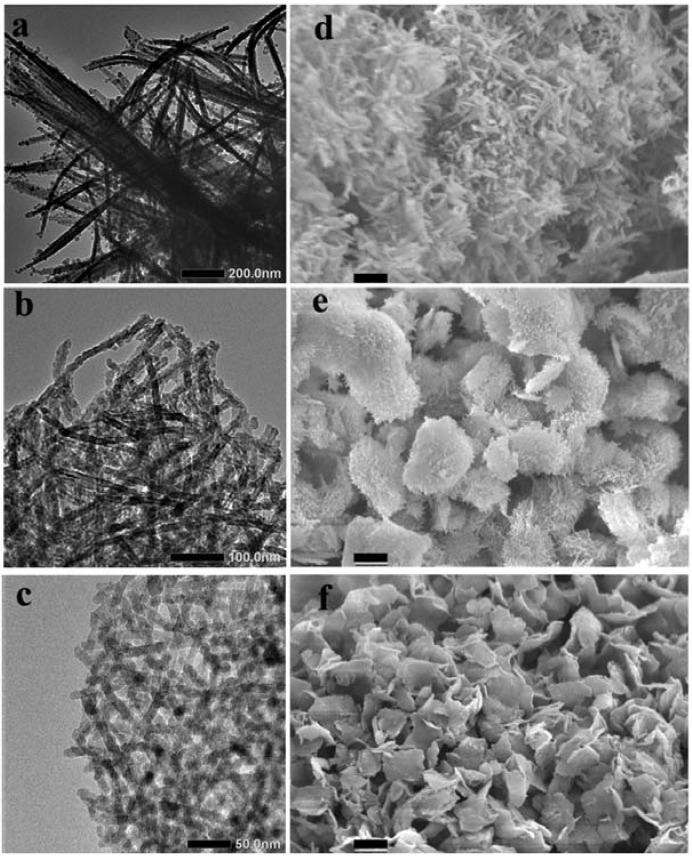
The transmission electron microscope (TEM) (left column) and scanning electron microscope (SEM) (right column) images of LPEI@SiO_2_ obtained under different cooling processes by fixing 10 g of 2 wt % LPEI and 10 mL of 10 vol % MS-51. From up row to bottom one, *m*_ice_ increased from 4, to 10, to 25 g, and corresponded to Process-I, -II, -III, respectively. Scale bars: (**d**), 1 μm; (**e**)–(**f**), 2.5 μm. Where LPEI was linear polyethyleneimine, MS-51 was methyl silicate-51.

Our first interest here is screening whether the crystalline LPEI aggregates generated by the differently accelerated cooling processes afford the specially structured silica after mixing the LPEI aggregates with MS-51 (methyl silicate-51, a commercially available silica source with 5-mer tetramethoxysilane). Therefore, we subjected the silica powders obtained from Process-I, -II and -III to TEM and SEM observation. Interestingly, as seen in the in Figure 1, the products of LPEI@SiO_2_ via different cooling processes showed different morphologies and sizes. In the case of Process-I, the produced LPEI@SiO_2_ appeared larger bundles in which a lot of one-dimensional nano structures (ribbon-like fibrils) coalesced each other. This is likely the case of natural cooling hot LPEI solution to room temperature in our previous reports [15]. Under the dramatically accelerated cooling conditions, however, the produced LPEI@SiO_2_ turned into the forms of nanotube-based platelets (in Process-II) and nanowires-based platelets (in Process-III). The size of the platelets in Process-II ranged from 5 to 10 µm but became small, below 5 µm, in Process-III. Interestingly, a lot of nanotubes with approximately 20 nm in diameter or nanowires approximately 10 nm in diameter knitted closely and densely each other forming platelets in which the nanotubes or nanowires grew nearly straightly towards the edge of platelets without embedding the ends in the platelets body. The nanotubes are approximately 3 nm hollow inside. In contrast, the silica mediated by the process-IV showed irregular fibrils-based bundles in which the fibrils are likely assemblies of irregular nanoparticles with an average size of 15 nm, *i.e.*, necklace-like structure (see Figure S1 in the Supporting Information). Therefore, it is conclusive that the morphologies and the fine structures of the 1D LPEI@SiO_2_ hybrids are easily controllable through different cooling processes.

Here, we can see a tendency towards the differences of 1D LPEI@SiO_2_ in nano-structure correlating to the morphologies variance in the micro-scale. This is attributed to the growth of the LPEI crystallites. In the previously mentioned three cooling process I-III, the final concentration of the LPEI and the lowest temperature after addition of ice decreased with increasing the amount of ice added. Despite an effect of quickly lowering temperature from above 85 °C to below 40 °C in Process-I, this quenching method did not affect to change the resulted silica structure compared to naturally cooling method in the conditions of the same concentration of LPEI. As seen in Figure 1d, the LPEI@SiO_2_ hybrids obtained in Process-I were mainly composed of bundles of fibers with diameters of several hundred nanometers and length of several micrometers. In our previous work, it was confirmed that the concentrations ranging 0.5~5 wt % LPEI resulted in the same ribbon-like fibrous silica bundles in the naturally cooling conditions [15]. Both in the Process-II and -III, the resulted silica powders have platelet-like morphologies, which are transcribed from LPEI precursors. This indicates that the suddenly quenched temperature arriving below 12 °C within 1 min is effective to change the growing direction of LPEI crystallites from bundles to platelets in which a lot of crystalline wires of LPEI crossly overlapped each other to form mat-like plates.

Since this biomimetic silicification included the pre-assembly of crystalline LPEI and post-deposition of silica, the amounts of LPEI and MS-51 may also impose control on the surface areas, porosity and compositions. Taking the Process-II as an example, we examined the silica deposition by changing the concentrations of LPEI and MS-51. In the TG-DTA analysis of the hybrids (see Figure S4 of TG-DTA charts), two endothermic peaks around 230–250 °C and 360–380 °C are observed, which can be attributed to the decompositions of LPEI. The mass ratios of silica/LPEI calculated based on the mass loss around 150~800 °C were listed in Table 2. It is evident that the LPEI@SiO_2_ hybrids resulted from higher concentrations of LPEI possess lower content of silica while the hybrids given upon higher concentrations of MS-51 trend to deposit plenty of silica.

**Table 2:** Composition of LPEI@SiO_2_ hybrids obtained from different conditions.

		**MS-51(in vol %)**		
		**10%**	**20%**	**30%**
		SiO_2_/LPEI	SiO_2_/LPEI	SiO_2_/LPEI
**LPEI concentration (wt %)**	**2**	77/23	82/18	81/19
	**5**	62/38	73/27	75/25
	**10**	54/46	65/35	68/32

**Figure 2:**
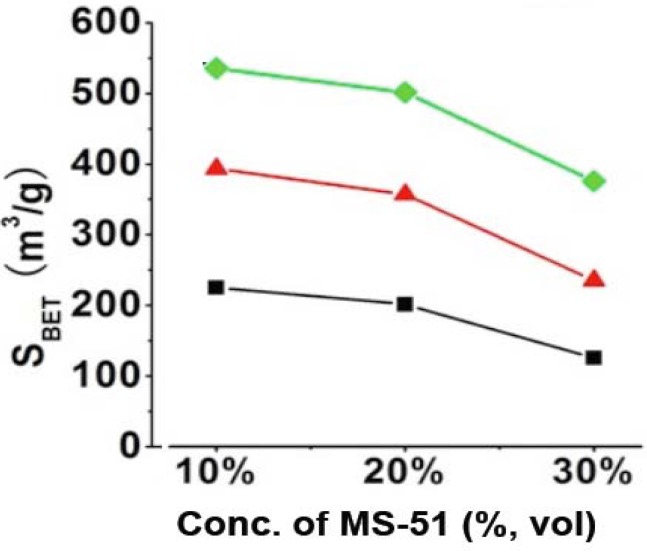
BET surface areas of SiO_2_ obtained by removing LPEI. Black line: 2% LPEI; Red line: 5% LPEI; Green line: 10% LPEI.

We calcined the LPEI@SiO_2_ hybrids at 800 °C and subjected the remained silica powders to N_2_ adsorption-desorption measurement to understand the characteristics in surface area and pore size distributions. It was found that the typical N_2_ adsorption-desorption isotherms for the calcined silica showed characteristic hysteresis loops as seen in porous materials and the pore size distribution ranged between approximately 5–30 nm (Figure S3). Such mesoporosity would be caused by the hollow structure of silica nanotube and the intergrain voids, which are constructed in the platelet piled up by a lot of 1D-structured nanosilica. It was found that the mesoporosity became smaller for the silica that was prepared under high concentrations of MS-51. Practically, the BET surface areas (S_BET_) largely related with the concentrations of LPEI (wt %) and MS-51 (vol %), which can be clarified in Figure 2. It is apparent that the values of S_BET_ for the silica elevated with increasing LPEI concentrations while declined with increasing MS-51 concentrations (Figure 2). Herein, the values of S_BET_ can be divided into three ranges based on LPEI concentrations: 100–200 m^2^/g for LPEI being 2.0%, 250–350 m^2^/g for 5.0% and 350–500 m^2^/g for 10% (in weight). Obviously, the higher concentration of LPEI was used, the larger surface area of silica was produced. This means that the LPEI enclosed in silica can play as a pore-directing agent; the more LPEI the more pores. On the other hand, S_BET_ decreased about 100 m^2^/g when concentration of MS-51 changed from 10% to 30% (in vol %), indicating that the relatively less amount of MS-51 are favorable to increase surface area of the silica. This is consistent with the increment of mesoporosity under lower concentration of MS-51. We confirmed that the SiO_2_ calcined at 800 °C, did not show morphological damage.

**Figure 3:**
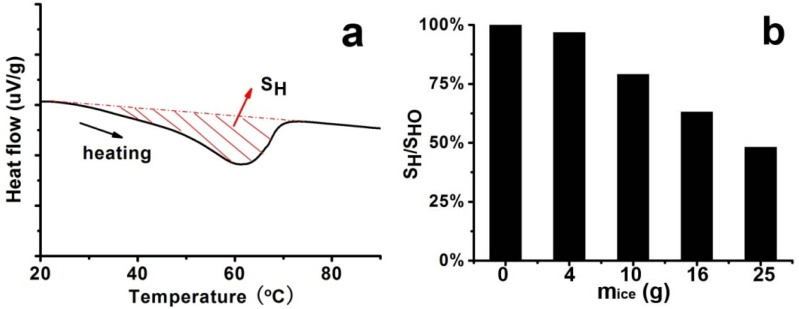
Differential scanning calorimetry (DSC) curve and integrated heat of fusion (S_H_) of samples under different cooling processes (different *m*_ice_) by fixing 5% LPEI. (**a**) Typical DSC curve; (**b**) Plot of ratios of S_H_/S_H__O_ against *m*_ice._

As pointed out by the results above, the 1D structure-based silica could be successfully modulated by the simple sudden cooling of the hot LPEI solution. The following issues are more about the details of the silicification process ranging from crystallization and assembly of LPEI to surface deposition of silica. Considering that the cooling was in fact a process of precipitation accompanying crystallization and rearrangement of LPEI chains, the nucleation and growth were hence interfered by the temperature change. For example, in Process-III with a strongly accelerated cooling process, the temperature suddenly reached 1 °C. This would cause a decrease of dissolved LPEI molecules in the solutions since LPEI is not soluble in water at ambient temperature condition. Because the formation of crystalline LPEI depends on the dissolved LPEI chains, which diffuse toward the as-formed nuclei, the growth and extension of the crystallites were then suppressed in this too quick cooling process. Consequently, the crystallization proceeded more rapidly with disordered fractions, compared with the long-range diffusion, and the nuclei were abundantly formed in a short time. In addition, in this case a part of LPEI molecules, which are independent of whether the thermodynamically most favored configuration is achieved, reached on the crystal surface and were incorporated into the crystal with amorphous state. As a result, short LPEI nano-wires with loose and irregular arrangements of LPEI chains were formed. This loose state allowed the permeation of silica source into the cores of LPEI nano-wires, leading to the formation of LPEI@SiO_2_ with solid appearance seen in TEM images. In Process II with an adequate cooling process, on the contrary, the crystallization proceeded more slowly compared with diffusion and the growth and rearrangement were supported, resulting in nano-fibers with rigid and regular arrangement of LPEI chains. This rigid structure is capable of blocking the permeation of silica source into the cores. In consequence, silica was mainly deposited on the surfaces to form silica shell. In this case, the TEM image just appeared as a tubular hollow structure. To some extent, the DSC data may further confirm the viewpoints above. The LPEI aggregates obtained via different cooling process were used for DSC analysis, and the typical heating curve was displayed in Figure 3a. The integrated heat of fusion (denoted as S_H_) on the heating curve was employed to judge the crystallinity [25,26]. By plotting the ratios of S_H_/S_HO_ against *m*_ice_ (where S_H_ is based the samples obtained under the conditions in the presence of ice and S_HO_ in the absence of ice), we compared the crystallinity of the LPEI aggregates. As seen in Figure 3b, the ratios declined with the increase of *m*_ice_, meaning that the crystallinity decreased. If the LPEI chains arranged irregularly and the crystalline LPEI backbone was short, less energy is needed for the melting of crystalline LPEI and the ratio of S_H_/S_HO_ would be estimated to be low. The ratio of S_H_/S_HO_ in Process-I (*m*_ice_ = 4 g) was about 97%, which indicates that our cooling process is near that in a natural cooling one. Indeed, the fibrous morphologies in the SEM are quite similar to our previous reports. But with increasing the ice amount (*i.e.*, decrease of S_H_/S_HO_), the evident morphological changes in the resulted silica happened. Although the detailed and complex assembly processes of crystalline LPEI are not clear, from the resulted silica structure, we can expect that in the accelerated cooling process the short and individual crystalline nano-wires of LPEI were geometrically favorable to link and knit each other growing to be the platelet-like or film-like morphologies. However, within the non-accelerated cooling system, the formed long nano-fibers of LPEI became difficult to assembly on a planar film, and finally presented as fibrous bundles as seen in SEM image.

**Figure 4:**
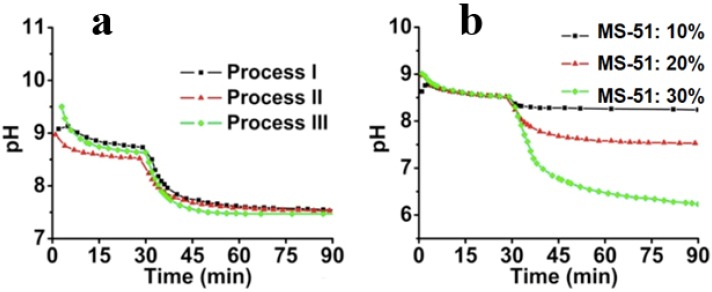
Time-course of pH change. (**a**) pH change curves under different cooling processes (different *m*_ice_) by fixing 5 wt % LPEI and 10 vol % MS-51; (**b**) pH change curves under different concentrations of MS-51 by fixing 10 wt % LPEI in Process-II (*m*_ice_ = 10 g).

The second issue to be discussed is that of the silica deposition process on the LPEI surfaces. After the organization and crystallization of LPEI in the first stage, it was suggested that the amorphous LPEI existing on the crystalline backbone promotes the hydrolytic condensation of MS-51. To monitor the deposition process, the dynamic pH change was recorded as shown in Figure 4. Here, the starting point to determine the pH value was the time of mixing the hot LPEI solution with crushed ice. During the initial 30 min, the hot LPEI solution cooled down to thermo-equilibrium state. At 30 min, MS-51 was added, and stirred for 60 min to complete the hydrolytic condensation reaction. Figure 4a shows the dynamic change of pH within different processes. It can be seen that after 15 min following the addition of ice, the pH began to plateau, indicating the closing of the crystallization. After the addition of MS-51, it also took approximately 15 min for the pH to become steady, meaning that the deposition of silica proceeded quickly to a large degree. In spite of the differences of *m*_ice_, the pH curves are fairly similar. In Figure 4b, the influence of MS-51 concentration on the pH was also investigated. Obviously, the final pH was correlated with concentrations of silica source. When MS-51 concentration was 30%, the final pH even closed to 6, indicating that high concentration of silica source led to low pH value.

The decline of pH offers us some information on LPEI-SiO_2_ interfaces. In the hot LPEI solution, because a lot of the –NH– functional groups in LPEI, which was dissolved molecularly, can accept protons from H_2_O, the hot solution should be relatively basic. After cooling, the LPEI self-assemble to aggregates and a limited amount of –NH– group will be localized on the surface of LPEI aggregates and forms an interface with water. This interface offers free OH^−^ in water phase so that pH value appears above 8.5. Choosing Process-II as a typical instant, we compared the influences of the concentration of MS-51 on the pH values in silica deposition. As seen in Figure 4b, after the addition of MS-51, the pH began to decrease with time. Here, the final pH after 90 min appeared at 8.4, 7.5 and 6.3, respectively, when MS-51 was 10%, 20% and 30%. To address the decline of pH, it is necessary to mention the mechanism of the amine group on LPEI aggregates in promoting the hydrolytic condensation of alkoxysilane. In fact, the groups of –NH– on LPEI aggregates play as catalysts for hydrolysis and condensation of alkoxysilane. During the progress of hydrolytic condensation, silica deposited around the exposed amine groups on the surface of LPEI aggregate and thus LPEI-H_2_O interface was quickly replaced by LPEI-SiO_2_. Consequently, the silica shell formed surrounding LPEI aggregates will neutralize the LPEI because the silica is weakly acidic, resulting in the pH decline. Figure 4b indicates unambiguously that when the mass of silica shell surrounding LPEI is low (*i.e.*, MS-51 concentration is low), the declined degree of pH is small. In contrast, as mass of silica deposited is high, the declined degree of pH is large (*i.e.*, the pH is lowered evidently). In addition, we can say that the silica deposition capacity become large when the concentration of LPEI was increased to 5%. In this case, 10% MS-51 (1mL of MS-51 in 9 mL ethanol) is insufficient for terminating the surface power to silica deposition.

**Figure 5:**
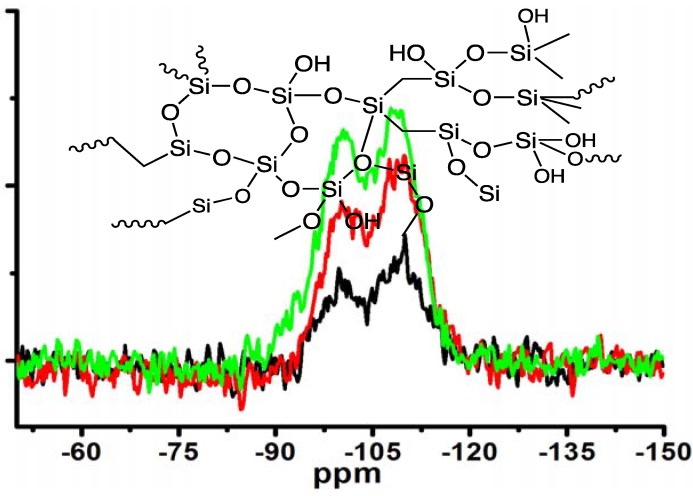
^29^Si CPMAS NMR spectra of the samples under different concentrations of MS-51 by fixing 10 wt % LPEI in Process-II (*m*_ice_ = 10 g). Insert: schematic description of amorphous silica. Black line: 10 vol % MS-51; Red line: 20 vol % MS-51; Green line: 30 vol % MS-51.

Moreover, the silica obtained under different concentrations of MS-51 while fixing LPEI concentration in Process-II was subjected to measurement of ^29^Si CPMAS NMR spectra (Figure 5). Two signals on the NMR spectra at −100 ppm and −110 ppm are assigned to Q3 (HOSi(OSi–)_3_) and Q4 (Si(OSi–)_4_), respectively. The estimated ratio of Q3/Q4 increased from 0.62, to 0.89, to 1.20 as the concentrations of MS-51 changed from 10, to 20 and to 30 vol %. Considering the hydrolytic condensation process of silica source, at the beginning the polycondensation grows towards branching dimension with a sufficient number of siloxane bonds (Si–O–Si) due to basicity of the surfaces of LPEI templates. However, as the mass of silica shell surrounding the LPEI aggregates increased, the basicity of its surface decreased. This basicity decline will suppress the branching growing of Si–O–Si but allow the polycondensation growing with linear fashion. Consequently, the Q4 bonding fraction decreased while Q3 bonding fraction increased when deposited silica increased (*i.e.*, at the higher concentration of MS-51).

The process of using the crushed ice to modulate the LPEI crystallization and subsequent silica deposition is quite convenient for scalable production of silica platelets with elemental one-dimension structures. For example, about 10 g of hybrid sample could be collected under the conditions as follows: LPEI (5 wt %, 100 g), 160 g of ice, MS-51 (30 vol %, 100 mL). These hybrids showed the sheet-like platelets appearance with nanowire-knitted structure inside (Figure S5).

**Figure 6:**
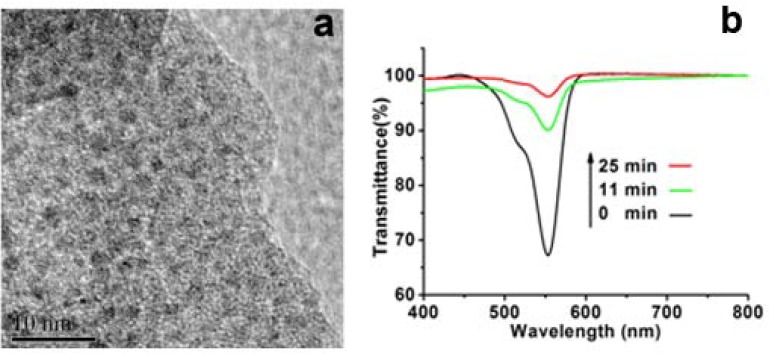
(**a**) High-resolution transmission electron microscopy (HRTEM) images of Pt-loaded LPEI@SiO_2_; (**b**) Time-course spectra of Rhodamine B (RhB) reduced by dimethylaminoborane (DMAB).

It is known that LPEI can act as a reducing agent and therefore the as-prepared LPEI@SiO_2_ can play both as reducing agent and host [19]. As an application, the LPEI@SiO_2_ hybrids obtained under the conditions of (Process-III, *m*_ice_ = 25 g, 5 wt % LPEI and 30 vol % MS-51) were employed to load Pt nanoparticles as catalyst. Here, just simply mixing PtCl_4_^2−^ solutions with LPEI@SiO_2_ powders, we loaded the Pt nanoparticles on the silica. As shown in Figure 6a, the HRTEM shows that Pt homogeneously distributed around the silica. The XRD pattern also shows the characteristic peaks of Pt (Figure S5). Since metallic nanoparticles can be used as catalysts in the organic reactions [27,28,29,30], we used the Pt-loaded SiO_2_ as a catalyst in the reduction of Rhodamine B by DMAB. As shown in Figure 6b, after mixing the catalyst of Pt-loaded SiO_2_ with the solution of containing RhB and DMAB, the absorption at around 553 nm of RhB decreased dramatically with almost de-colored state only within 11 min. In contrast, the solution without addition of the Pt-loaded SiO_2_ showed little color change even after 24 h. This contrast indicates that the Pt-loaded SiO_2_ is an active catalyst for the reduction of RhB by DMAB. As for the catalytic mechanism, the Pt nanoparticles loaded on silica wire can play as electron pool where the electrons from DMAB are trapped on Pt and then passed to RhB.

## 3. Experimental Section

### 3.1. Typical Synthesis of LPEI@SiO_2_ Hybrids

The preparation of LPEI (average molecular weight ~20000) was previously reported [15]. To 10 g of LPEI solution (5 wt %), which was heated to 85 °C in advance, was added immediately 10 g of crushed ice under magnetic stirring. This led to a dramatic decrease of the solution temperature to about 12 °C in 1 min and resulted simultaneously in aggregates of LPEI to form a suspension. After 30 min, the suspension temperature rose to room temperature. Then, to this suspension 10 mL of 10 vol % of methyl silicate-51 (MS-51 a commercially available silica source with 5-mer tetramethoxysilane) in ethanol was added. This mixture was stirred for 1 h at room temperature. Finally, the precipitates were collected by centrifugation, washed by ethanol, and dried at 60 °C under vacuum for 24 h.

In the present work, the concentrations of LPEI were adjusted to 2, 5 and 10 wt % before the addition of crushed ice. Three typical accelerated cooling processes were selected according to the amount of ice (denoted as *m*_ice_: 4 g, 10 g, and 25 g) and a temperature detector monitored the temperature change. Under each process, the concentrations of LPEI solution (10 g) with 2, 5 and 10 wt % and the volume concentrations of MS-51 in ethanol solution (10 mL) with 10, 20 and 30 vol % were also considered as control factors. To get silica, LPEI in the hybrids was removed by calcination at 800 °C for 3 h after a heating process of 5 h from room temperature to 800 °C.

### 3.2. Loading Pt Nanoparticles on LPEI@SiO_2_ Hybrids

0.04 g of LPEI@SiO_2_ powders obtained under the condition of [*m*_ice_ = 25 g, 2 wt % LPEI (10 g), 10 vol % MS-51 (10 mL)] were mixed with 10 mL of Na_2_PtCl_4_ solution (0.02 M). After stirring for 30 min under room temperature, the solution was shaken at 85 °C for 30 min. Then the Pt loaded LPEI@SiO_2_ powders (Pt-LPEI@SiO_2_) were obtained by centrifugation. To evaluate the catalytic activity, 10 mg of Pt-LPEI@SiO_2_ were mixed with 2 mL of Rhodamine B (RhB) (8 ppm) and 7 mL of H_2_O. After a stirring of 30 min, 1 mL solution containing the reducing agent of DMAB (dimethylaminoborane, (CH_3_)_2_NH·BH_3_, 0.2 mol/L) was added to the mixture, and the absorption spectra of RhB in supernatant was monitored using a UV-Vis spectrometer.

### 3.3. Characterizations

The morphologies of the products were observed by SEM (Kyence, VE9800) and TEM (JEOL, JEM-2200FS). The thermal decomposition behaviors were monitored by TG/DTA (SII Nano technology Inc., Japan, TG-DTA 6300). The BET surface areas were obtained from N_2_ absorption-desorption experiments performed on a Flow Sorb II 2300 Instrument (Micromeritics). The XRD patterns were recorded on a diffractometer (Rigaku-Denki RX-7) with CuK_α_ radiation. The polycondensation degree of silica framework was estimated by the ^29^Si CPMAS NMR spectra recorded on a JEOL-400 MHz NMR spectrometer. The melting behavior of LPEI aggregates under different cooling rates was monitored by DSC measurements (Perkin-Elmer DSC-6) with a heating rate of 10 °C/min. The absorption spectra of Rhodamine B were recorded on Hitachi U-4100 spectrophotometer.

## 4. Conclusions

In summary, a novel and scalable approach to regulate the structures and morphologies of silica was reported. Through the control of the crystallization and assembly of LPEI by simply changing cooling process with crushed ices, we can prepare tunable templates that are able to lead site-selectively to multiple morphologies of silica in a broad-scale length with nanofibers, nanotube and nanowire skeletons. Alongside the variation of the amounts of LPEI and silica source, the compositions and surfaces areas of LPEI@Silica can be further tuned. The results help us not only understand the specific roles of the three factors but also elucidate the silica deposition kinetic process. Using this biomimetic method, nanostructured silica with diverse patterns guided by LPEI can be easily and scalably available. As an example for application of the silica, Pt nanoparticles were loaded on the as-prepared silica, which showed a good catalysis for the reductions of RhB by DMAB.

## Supplementary material

Supplementary File 1
