# Supplementary material for: Polyamine-Promoted Growth of One-Dimensional Nanostructure-Based Silica and Its Feature in Catalyst Design
Source: Materials (Basel). 2012 Oct 1;5(10):1787–99. doi: 10.3390/ma5101787 (PMC5449029; doi:10.3390/ma5101787)
Supplement: Supplementary File 1 [file materials-05-01787-s001.pdf]

Supporting Information

Figure S1. TEM images of LPEI@SiO_2 obtained via process-IV with the conditions of 10 g of 5 wt % LPEI, 50 g of crushed ice and 10 mL of 20 vol % MS-51.

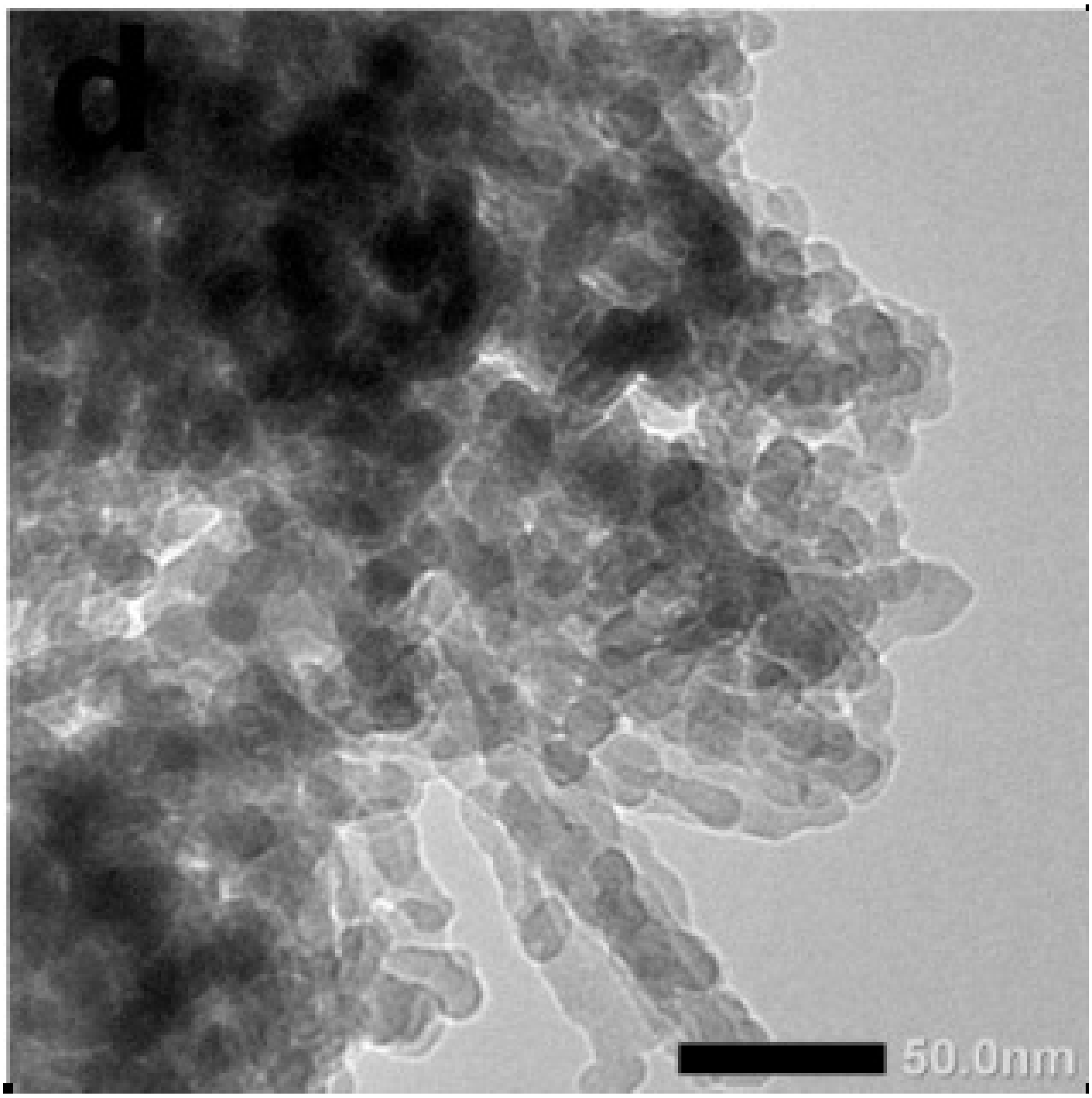

Figure S2. SEM images of LPEI@SiO_2 obtained under different concentrations of MS-51 (10 mL) by fixing 10 g of 2 wt % LPEI in Process-II (m_{ice} =10 g). (**a**) 10 vol %; (**b**) 20 vol %, (**c**) 30 vol %. Scale bars: 2.5 μm.

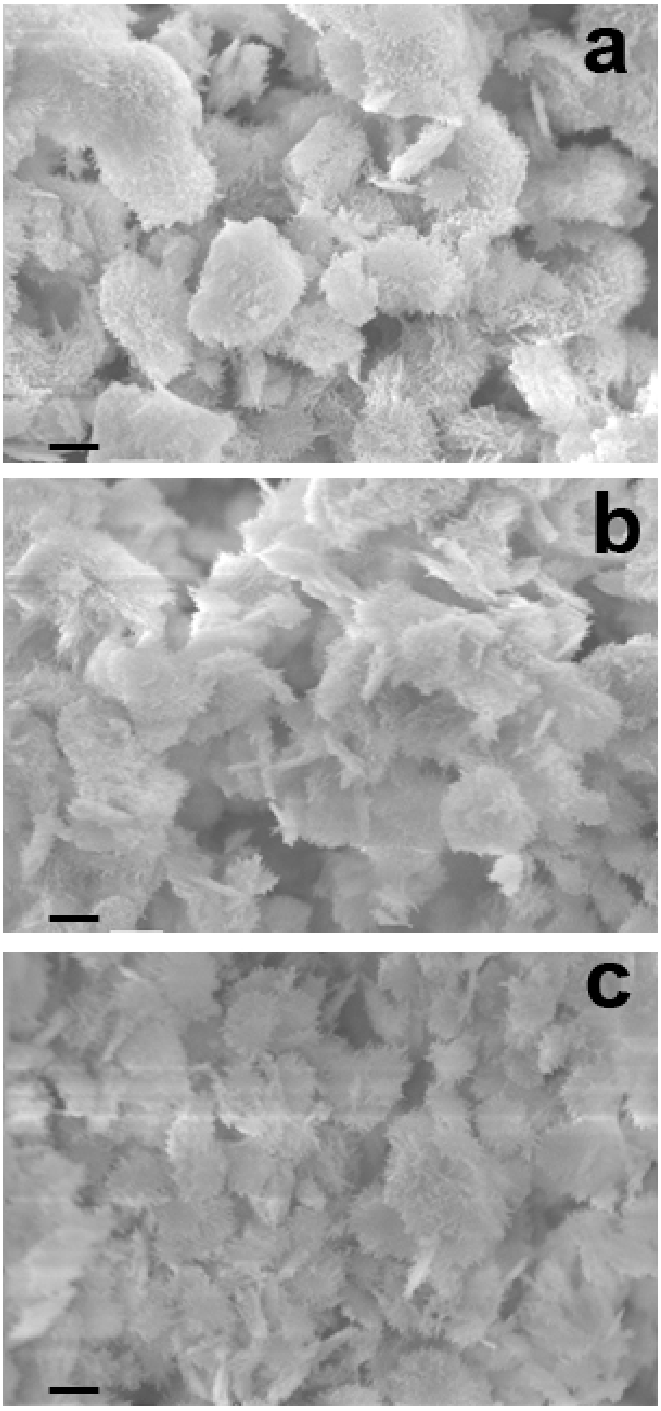

Figure S3. N_2 absorption-desorption isotherms of the calcined silica and the corresponding pore sizes distribution curves (by BJH, inserts) under different concentrations of LPEI and MS-51 in Process-II (m_{ice} = 10 g). (**a**) 2 wt % LPEI; (**b**) 5 wt % LPEI; (**c**) 10 wt % LPEI. Black line: 10 vol % MS-51; Red line: 20 vol % MS-51; Green line: 30 vol % MS-51.

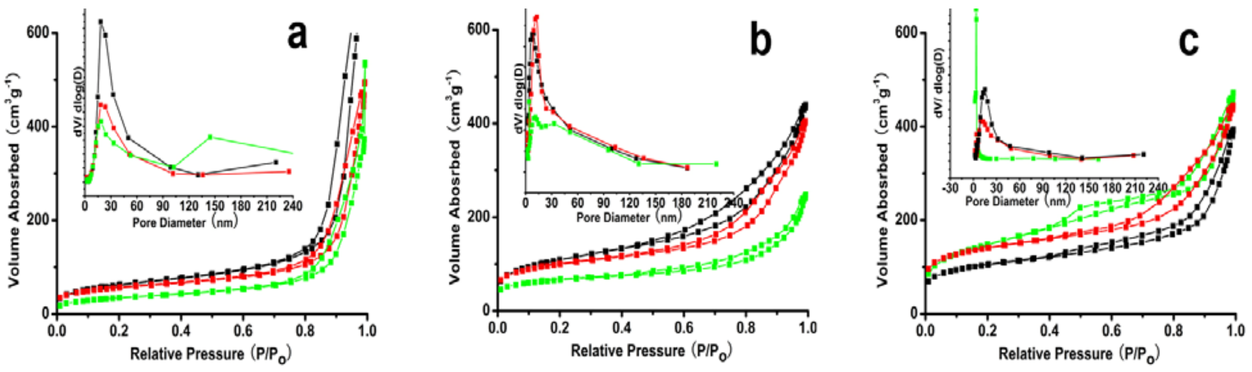

Figure S4. TGDTA curves of LPEI@SiO_2 obtained under different concentrations of MS-51 by fixing 2 wt % LPEI in Process-II (m_{ice} = 10 g). Black line: 10 vol %; Red line: 20 vol %; Green line: 30 vol %.

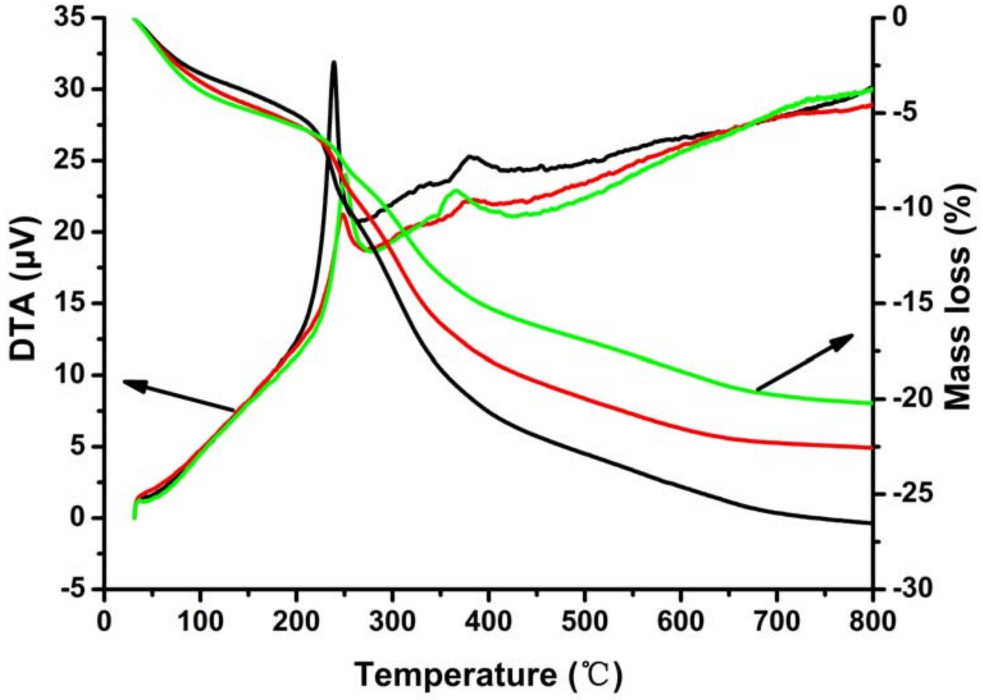

Figure S5. The SEM image (left, scale bar: 2.5 μm) and TEM image (right) of the sample obtained under the conditions: m_{ice} = 160 g, 100 g of 5 wt % LPEI solution, 100 mL of 30 vol % MS-51 solution.

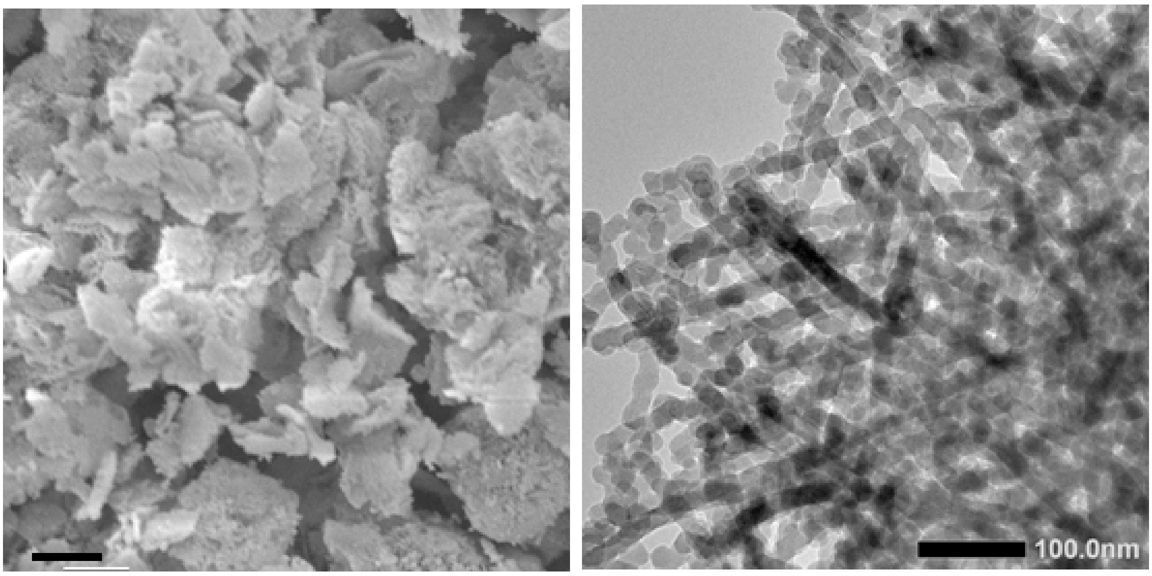

Figure S6. The X-ray powder diffraction (XRD) pattern of the sample of Pt loaded LPEI@SiO_2.

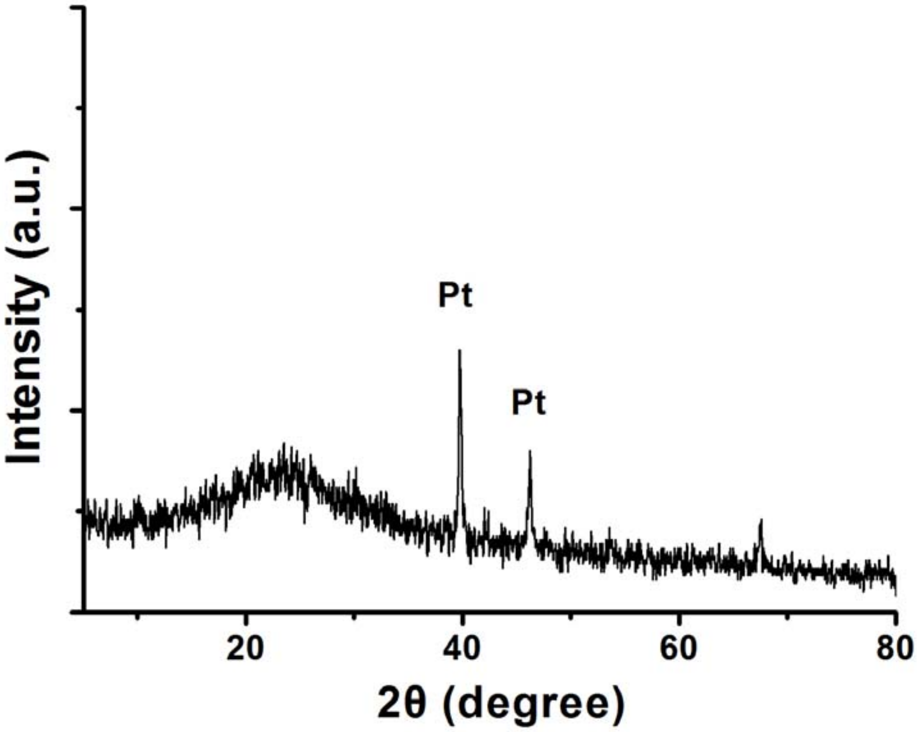

© 2012 by the authors; licensee MDPI, Basel, Switzerland. This article is an open access article distributed under the terms and conditions of the Creative Commons Attribution license (http://creativecommons.org/licenses/by/3.0/).
